# Transcriptome Profiling of Buffalograss Challenged with the Leaf Spot Pathogen *Curvularia inaequalis*

**DOI:** 10.3389/fpls.2016.00715

**Published:** 2016-05-25

**Authors:** Bimal S. Amaradasa, Keenan Amundsen

**Affiliations:** ^1^Department of Plant Pathology, University of Nebraska–Lincoln, LincolnNE, USA; ^2^Department of Agronomy and Horticulture, University of Nebraska–Lincoln, LincolnNE, USA

**Keywords:** buffalograss (*Bouteloua dactyloides*), *Curvularia inaequalis*, leaf spot, defense-related genes, transcriptome, next-generation sequencing

## Abstract

Buffalograss (*Bouteloua dactyloides*) is a low maintenance U. S. native turfgrass species with exceptional drought, heat, and cold tolerance. Leaf spot caused by *Curvularia inaequalis* negatively impacts buffalograss visual quality. Two leaf spot susceptible and two resistant buffalograss lines were challenged with *C. inaequalis*. Samples were collected from treated and untreated leaves when susceptible lines showed symptoms. Transcriptome sequencing was done and differentially expressed genes were identified. Approximately 27 million raw sequencing reads were produced per sample. More than 86% of the sequencing reads mapped to an existing buffalograss reference transcriptome. *De novo* assembly of unmapped reads was merged with the existing reference to produce a more complete transcriptome. There were 461 differentially expressed transcripts between the resistant and susceptible lines when challenged with the pathogen and 1552 in its absence. Previously characterized defense-related genes were identified among the differentially expressed transcripts. Twenty one resistant line transcripts were similar to genes regulating pattern triggered immunity and 20 transcripts were similar to genes regulating effector triggered immunity. There were also nine up-regulated transcripts in resistance lines which showed potential to initiate systemic acquired resistance (SAR) and three transcripts encoding pathogenesis-related proteins which are downstream products of SAR. This is the first study characterizing changes in the buffalograss transcriptome when challenged with *C. inaequalis*.

## Introduction

Buffalograss (*Bouteloua dactyloides*) is a U. S. native, warm-season turfgrass species with exceptional drought, heat, and cold tolerance (Beetle, 1950; Reeder, 1971). Buffalograss requires less fertility, pesticides and water to maintain an acceptable quality level compared to traditional turfgrass species (Riordan et al., 1993). Replacing traditional turfgrass species with buffalograss may help to conserve water, especially in the semi-arid and arid regions of the USA (Riordan et al., 1993). Buffalograss is tolerant of many diseases, but leaf spot can cause decline or death of buffalograss turf. Leaf spot is caused by several species belonging to the *Curvularia, Bipolaris*, and *Cercospora* genera (Smith et al., 1989; Smiley et al., 2005). In Nebraska, *Curvularia inaequalis* (Shear) Boedijn and *Bipolaris spicifera* (Bainier) Subram (teleomorph: *Cochliobolus spicifer* Nelson) are commonly isolated from buffalograss with leaf spot symptoms (Amaradasa and Amundsen, 2014b). On lawns, leaf spot initiates as dark brown leaf spots followed by leaf tip dieback and eventual blighting of entire tillers. As the disease progresses, patches of leaf decline and canopy thinning occur. Leaf spot symptoms of *C. inaequalis* and *B. spicifera* are identical and therefore it is not possible to distinguish the causal organism by disease symptoms alone. Disease development commonly occurs when temperatures are 30°C and above. Disease severity increases when buffalograss is under stress by adverse weather conditions such as high temperatures, high humidity, drought, excess rain, and cloud cover. Since buffalograss is often considered a low-maintenance turfgrass, the use of fungicides is usually not preferred by homeowners and lawn care managers. Incorporating host resistance through plant breeding is one way to combat leaf spot disease. Conventional breeding for disease resistance is difficult and time consuming, and is based on inoculation, rating for incidence and severity of disease, and selection of resistant genotypes. Identification of genes that confer leaf spot resistance would enable molecular-based strategies to improve the efficiency of breeding for resistant cultivars.

Today, comparative genetic studies using next generation sequencing (NGS) technology are common for characterizing gene functions in plants and other organisms. NGS technology can be used to sequence both genomic DNA and total RNA (RNA-seq). The large number of short sequencing reads produced by this technology is highly cost effective and can be used to assemble a genome or transcriptome *de novo* or can be mapped to a reference to determine differentially expressed genes. Buffalograss has a basic chromosome number of 10 and exists as a ploidy series of diploids, tetraploids, pentaploids, and hexaploids (Johnson et al., 1998). This large repetitive genome makes whole genome sequencing and annotation difficult. Conversely, transcriptome profiling by RNA-seq has been used to decipher differentially expressed genes in grass systems (Wang et al., 2009; Gutierrez-Gonzalez et al., 2013; Wachholtz et al., 2013; Yang et al., 2013). The number of short-reads from RNA-seq data gives an indication of the level of gene expression and therefore is highly suitable for gene expression studies (Wang et al., 2009).

To identify differentially expressed defense-related genes in buffalograss, we profiled transcriptomes of two leaf spot resistant and two susceptible lines after challenging with *C. inaequalis*. For this study, we chose to use *Curvularia* over *Bipolaris* since it is more virulent and produces disease symptoms faster. *De novo* assembly of RNA-seq data from a previous Prestige buffalograss NGS study resulted in a reference assembly of 91,519 contigs (Wachholtz et al., 2013); this previously published buffalograss transcriptome was used as a reference in our study. In the previous study, basal transcriptional expression differences were compared between the two buffalograss cultivars Prestige and 378. However, identification of defense-related genes in response to a pathogen was not part of the previous study. The main objective of our study was to identify buffalograss leaf spot resistance genes differentially expressed between resistant and susceptible buffalograss.

## Materials and Methods

### Buffalograss Inoculation and Leaf Tissue Sampling

Two leaf spot resistant (95-55 and NE-BFG-7-3459-17) and two susceptible buffalograss lines (Prestige and NE-BFG-7-3453-50) identified previously were used in our study (Amaradasa and Amundsen, 2014a). Stolons of leaf spot resistant lines and susceptible lines were planted in 7-cm-diameter plastic pots filled with Fafard^®^ 3B Mix potting medium. Pots were kept in a greenhouse with a 16 h day and 8 h night photoperiod. The average daytime and nighttime temperature of the greenhouse was maintained at 30 and 22°C, respectively. Plants were watered daily, fertilized biweekly with 20–20–20 to provide an approximate annual rate of 10 g N m^-2^, and clipped with scissors regularly to a height of 6 to 7 cm to promote prostrate growth and pot coverage. After 12 weeks of growth, plants were arranged in a randomized complete block design (RCBD) with three replications. Single-spore *C. inaequalis* strain 4L-SS01 was used to prepare a spore culture of 1 × 10^6^ spores ml^-1^ according to published methods (Brecht et al., 2007). Each pot was sprayed with 15 ml of the spore solution. Untreated controls were also included with three replicates and sprayed with water in place of the spore solution. After 10 days, when susceptible lines were exhibiting distinct disease symptoms, leaf tissue from both inoculated and uninoculated pots was harvested into separate freezer bags and immediately frozen in liquid nitrogen. Samples were kept at -80°C for later use.

### Total RNA Sequencing and Analysis

Approximately 100 mg of leaf tissue of each sample (95-55, NE-BFG-7-3459-17, Prestige, and NE-BFG-7-3453-50) was homogenized in liquid nitrogen using a mortar and pestle and RNA was extracted using an RNeasy Plant Mini Kit (Qiagen, Valencia, CA, USA) according to the manufacturer’s instructions. RNA samples were qualitatively analyzed by agarose gel electrophoresis, and quantified using a NanoDrop 2000C spectrophotometer (Thermo Fisher Scientific Inc., Wilmington, DE, USA). Total RNA from 24 samples [4 buffalograss lines × 3 replicates × 2 treatments (inoculated/uninoculated)] was sent to the High-Throughput DNA Sequencing and Genotyping Core Facility located at the University of Nebraska Medical Center, Omaha, Nebraska for transcriptome sequencing. The cDNA libraries were prepared and then sequenced using a HiSeq 2000 sequencing platform (Illumina, San Diego, CA, USA) according to the manufacturer’s RNA-seq protocol. The 24 samples were separately barcoded and run on three lanes of the HighSeq 2000 to obtain 100 bp single-end reads. Quality filtering of the reads was done by the Genotyping Core Facility. FastQC^1^ was used to visualize the quality of the reads using default parameters. Since FastQC showed several overrepresented reads consisting of Illumina adapter and primer sequences, Trimmomatic-0.30 (Bolger et al., 2014) was used to remove those contaminants. The reads were trimmed to a uniform length of 80 bp prior to downstream analysis. A fastq file containing the sequencing reads and quality data was used for down-stream analysis. The sequencing reads were mapped with Bowtie2-2.1.0 (Langmead and Salzberg, 2012) to the *B. dactyloides* cv. Prestige transcriptome (Wachholtz et al., 2013). Reads that did not map to the reference were retained and assembled using Trinity-r2013-02-25 (Grabherr et al., 2011). The Trinity assembled contigs and the Prestige reference transcriptome were merged and cd-hit-est version 4.5.4 (Weizhong and Godzik, 2006) was used to remove redundancy with a 100% identity threshold to create the buffalograss transcriptome. Single-end raw sequencing reads of each individual were mapped with Bowtie2 to the buffalograss transcriptome to allow for the estimation of transcript abundance per individual relative to the buffalograss transcriptome. A read count table was produced using SAMtools (Li et al., 2009) and Perl^2^. To account for the variability of total initial Illumina sequencing results among samples, mapped read counts were subjected to normalization and then analyzed for differential expression using the DESeq2 Bioconductor package (Love et al., 2014) in R program (version 3.0.2).

Read counts of the two inoculated resistant (R) lines were compared separately to each inoculated susceptible (S) line. We used a final adjusted *P*-value of < 0.01 to select transcripts that showed a difference in expression between inoculated resistant and susceptible lines. The differentially up-regulated transcripts of 95-55 (R) vs. NE-BFG-7-3453-50 (S) were compared with 95-55 (R) vs. Prestige (S) and common transcripts were identified. Similarly, common up-regulated transcripts between inoculated resistant line NE-BFG-7-3459-17 (R) vs. inoculated susceptible lines were selected. Then the two sets of up-regulated genes (i.e., 95–55 vs. susceptible lines and NE-BFG-7-3459-17 vs. susceptible lines) were compared to each other and common transcripts were identified for annotation. By this filtering procedure, we identified transcripts that are common and differentially up-regulated in both inoculated resistant lines compared to the susceptible lines (up-regulated in resistant inoculated; URI). In the same way, we compared each uninoculated resistant line with each uninoculated susceptible line and used the same filtering procedure to identify genes in common that have different levels of expression between both resistant and susceptible lines (basal up-regulated expressions; BUE). Then we identified down-regulated transcripts in resistant inoculated and uninoculated cultivars compared to inoculated and uninoculated susceptible lines, respectively (down-regulated in resistant inoculated: DRI; basal down-regulated expressions: BDE).

The four sets of transcripts were pooled and annotated with Blast2GO using default settings (Conesa et al., 2005). The annotated transcripts were analyzed separately to identify genes responsible for induced resistance (transcripts of URI and DRI) and innate immunity (transcripts of BUE and BDE) in buffalograss. Blast2GO was used to prepare graphs of biological, cellular and molecular processes at level two gene ontologies. Gene ontology IDs resulting from the analysis were mined for disease resistance related terms.

### Validation of BUE and URI Gene Expression

Ten differentially expressed transcripts were selected for validation by reverse transcription PCR (RT-PCR). These transcripts showed up-regulation either in inoculated or uninoculated resistant buffalograss lines compared to inoculated or uninoculated susceptible lines, respectively. Some transcripts were chosen because they did not have any read counts for either inoculated or uninoculated susceptible lines (Supplementary Table S1). The primers (**Table 1**) were synthesized for each transcript using Primer3web^3^ version 4.0.0. The primers for ubiquitin conjugating enzyme (*UCE*) were used as a positive control (**Table 1**). RNA was extracted from new plants of inoculated and uninoculated 95–55, NE-BFG-7-3459-17, Prestige, and NE-BFG-7-3453-50 as described previously. cDNA was synthesized using an Invitrogen^TM^ SuperScript^®^ III First-Strand Synthesis System (Life Technologies, Grand Island, NY, USA) according to the manufacturer’s instructions. Using a standard PCR protocol (each 25 μl reaction mixture contained 1x Taq DNA polymerase buffer, 0.2 μM forward and reverse primers, 0.2 mM each dNTPs, 1 U Taq polymerase) each primer pair was used to amplify 100 ng of cDNA template in a Mastercycler^®^Pro thermalcycler (Eppendorf, Hamburg, Germany) with the following conditions: Initial denaturation at 94°C for 3 min, was followed by 35 cycles at 94°C for 30 s, 55°C for 1 min, and 72°C for 1 min, and a final extension at 72°C for 10 min. Thereafter the reaction was stopped by reducing the temperature to 4°C and PCR products were stored at -20°C. Aliquots (5 μl) of amplified products were separated by electrophoresis on a gel containing 1.7% (w/v) agarose and 1x TAE, at 100 V for 1 h. The presence and size of the DNA fragments were verified by staining the gel with ethidium bromide and observing under UV light.

**Table 1:** Primers used for validating expression of selected buffalograss transcripts.

Transcript name	Transcript length	Forward primer (5′–3′)	Reverse primer (3′–5′)	PCR product size
BG_UCE^a^		GACCCGCCTACATCATGCA	TTGCTTGCCAGTGAAACATGTC	61
Bodac2015c080259	258	CCAGCAAGTTCGAGAGATCC	GGCACTGCCGGAATGTATAA	203
Bodac2015c126512	223	AAAATCAGGGGATCCCAAAC	GGAAGTGCTTGGCCTAGGTA	176
Bodac2015c127986	226	AGCCTCTGCAAACCAAGAAA	CCGCAAGCTTAACCTTCATC	158
Bodac2015c166783	1483	AGCACTACTCTGCGTCAGCA	CGTCATTGCTATGGATGGTG	194
Bodac2015c163663	2499	AAACCACGGAAGCCTTTTCT	GCATGTGCATTTTGTTTTGG	489
Bodac2015c100000	563	GATAGGCGTGTGAAACAGGAA	CAGTGTGGACACCTTGGTTG	423
Bodac2015c000129	1874	GCACAGGCCGTACAATACCT	CGACTGACGACCCGAATAAT	210
Bodac2015c064306	317	GACGCAGTGAGGATCAGTTG	GCCATGCGTTTCTACTCTCC	164
Bodac2015c084994	414	TTTGATGATCGGTGCATCTC	GCCTCCATCTCATCCTCTTG	249
Bodac2015c087283	501	AAAGGCAACTCATCGACCTC	CAACCTTGCGTTCATTTGC	216

*^a^Endogenous control ubiquitin conjugating enzyme gene.*

## Results

### Filtering and *De Novo* Assembly of Raw Reads

**FIGURE 1:**
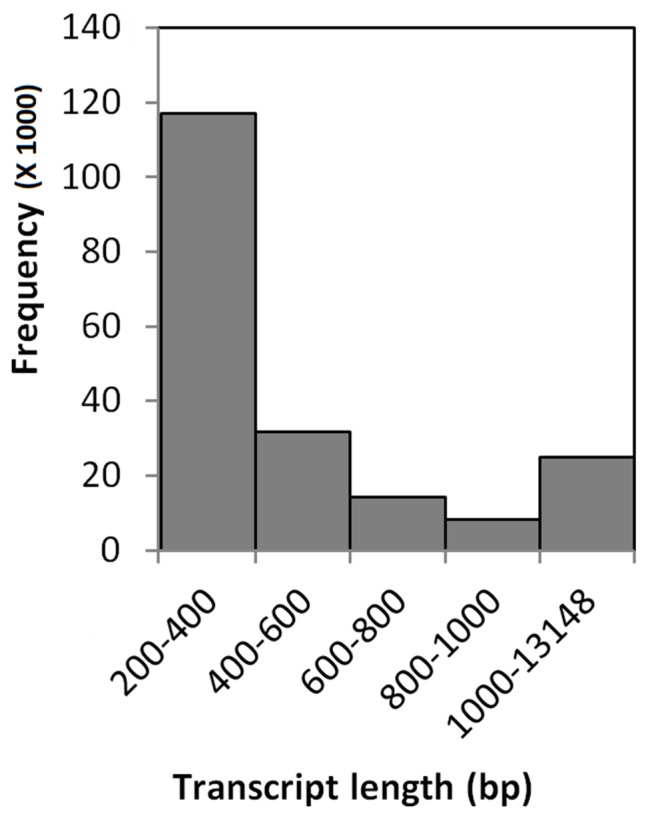
**Transcript length frequency distribution of the final buffalograss reference transcriptome, consisting of 196168 transcripts**.

Sequencing of twenty four cDNA libraries constructed from *C. inaequalis* infected as well as non-infected buffalograss lines produced approximated 655.2 million 100 bp single-end reads (**Table 2**). On average, 30.5 million reads were produced per sample from inoculated leaf tissue and 24.0 million reads were produced per non-inoculated leaf tissue sample. Trimmomatic removed 9 to 17% of overrepresented sequences (**Table 2**). The existing *B. dactyloides* cv. Prestige reference transcriptome (Wachholtz et al., 2013) had 91,519 contigs. When raw reads of each of the buffalograss samples were mapped to the Prestige reference, 2 to 14% of the reads did not map (**Table 2**). The unmapped raw reads were assembled by Trinity into 156,721 contigs. These contigs were merged with the existing Prestige reference resulting in the creation of the buffalograss transcriptome which consisted of 248,221 transcripts. The final buffalograss reference used for read mapping was prepared by removing all isoforms and keeping only the longest transcript for each gene. This final buffalograss reference had 196,168 transcripts with the longest transcript having 13,148 bp, average transcript length of 565 bp, and median transcript length of 340 bp. We submitted the sequences of final reference to NCBI BioProject repository^4^ under the project ID PRJNA297834. The distribution of transcript lengths is depicted in **Figure 1**. When the sequencing reads from the 24 buffalograss samples were mapped to the final buffalograss reference, more than 98% of the reads mapped at least once (**Table 2**).

**Table 2:** Summary of raw reads per sample, quality filtering, and mapping percentages of surviving reads.

Buffalograss samples^a^	Total raw reads (millions)	Total reads after removing contaminants (millions)	Surviving reads mapped to Prestige reference^b^ (%)	Surviving reads mapped to final buffalograss reference (%)
17C1	24.4	21.9	97.01	99.10
17C2	26.5	24.1	97.25	99.23
17C3	31.5	28.3	97.6	99.38
17T1	27.6	24.3	94.44	99.06
17T2	33.3	29.8	94	98.97
17T3	32.3	28.6	92.91	98.93
50C1	25.8	23.1	96.83	99.13
50C2	19.6	17.6	97.05	99.23
50C3	26.8	23.7	97.14	99.27
50T1	20.3	17.1	92.81	98.58
50T2	58.1	49.0	94.47	99.11
50T3	20.9	18.0	94.99	99.14
95C1	21.9	19.4	97.56	99.31
95C2	21.6	19.5	97.47	99.34
95C3	24.0	21.7	98.08	99.39
95T1	33.0	27.9	94.92	99.16
95T2	28.7	24.3	95.11	99.15
95T3	30.9	27.4	94.42	99.06
PC1	18.7	16.6	90.71	99.06
PC2	23.5	20.4	86.57	98.78
PC3	24.6	22.0	92.37	99.07
PT1	27.2	23.3	90.18	98.57
PT2	25.0	20.8	94.8	99.11
PT3	29.0	24.5	95.89	99.13

*^a^17, 50, 95 and P refers to NE BG 7-3459-17, NE BG 7-3453-50, 95-55 and Prestige, respectively. C and T indicate untreated and treated samples. ^b^Wachholtz et al. (2013).*

### Identification of Differentially Expressed Transcripts

**FIGURE 2:**
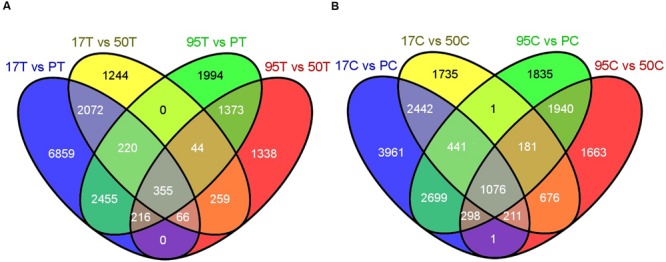
**(A)** Number of differentially up-regulated (DUR) transcripts in inoculated resistant lines (17T and 95T) compared to the inoculated susceptible lines (PT and 50T). **(B)** Number of DUR transcripts in uninoculated resistant lines (17C and 95C) compared to the uninoculated susceptible lines (PC and 50C). 17, 50, 95 and P refer to NE BG 7-3459-17, NE BG 7-3453-50, 95-55 and Prestige, respectively. C and T indicate untreated and treated samples, respectively. The Venn diagrams were generated by the Venny tool (http://bioinfogp.cnb.csic.es/tools/venny/) using the DUR transcripts resulting from DESeq2 by comparing each resistant and susceptible line.

**FIGURE 3:**
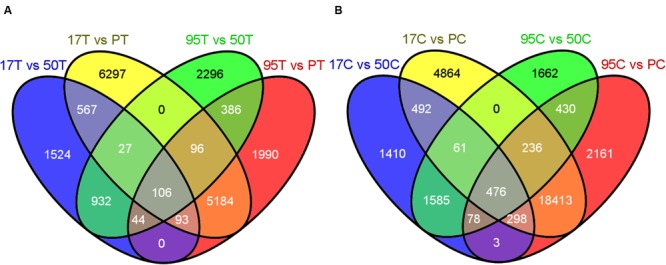
**(A)** Number of differentially down-regulated (DDR) transcripts in inoculated resistant lines (17T and 95T) compared to the inoculated susceptible lines (PT and 50T). **(B)** Number of DDR transcripts in uninoculated resistant lines (17C and 95C) compared to the uninoculated susceptible lines (PC and 50C). 17, 50, 95 and P refer to NE BG 7-3459-17, NE BG 7-3453-50, 95–55 and Prestige, respectively. C and T indicate untreated and treated samples, respectively. The Venn diagrams were generated by the Venny tool (http://bioinfogp.cnb.csic.es/tools/venny/) using the DDR transcripts resulting from DESeq2 by comparing each resistant and susceptible line.

The DESeq2 analysis of inoculated lines resulted in 355 up-regulated transcripts (URI) and 106 down-regulated transcripts (DRI) in both resistant lines when compared to the susceptible lines (**Figures 2A** and **3A**). Similarly, the uninoculated resistant lines had 1,076 transcripts with higher expression (BUE) and 476 transcripts with lower expression (BDE) in common when compared with the uninoculated susceptible lines (**Figures 2B** and **3B**). There were 75 transcripts in common among the two up-regulated transcript sets (URI and BUE) and 14 common transcripts in the two down-regulated transcript sets (DRI and BDE). Eight URI transcripts had more than 10 mapped reads for each of the resistant samples and no mapped reads for the susceptible samples. Of these eight transcripts, five were in common with the BUE transcripts. Similarly, nine BUE transcripts had at least 10 mapped reads for each of the resistant samples and no mapped reads for the susceptible samples. Of these nine transcripts, two were in common with the URI transcripts. Two transcripts (Bodac2015c153835 and Bodac2015c154561) of DRI group had no mapped reads for any of the resistant samples while all of the inoculated susceptible samples had more than 10 mapped reads.

### Annotation of Differentially Expressed Transcripts

**FIGURE 4:**
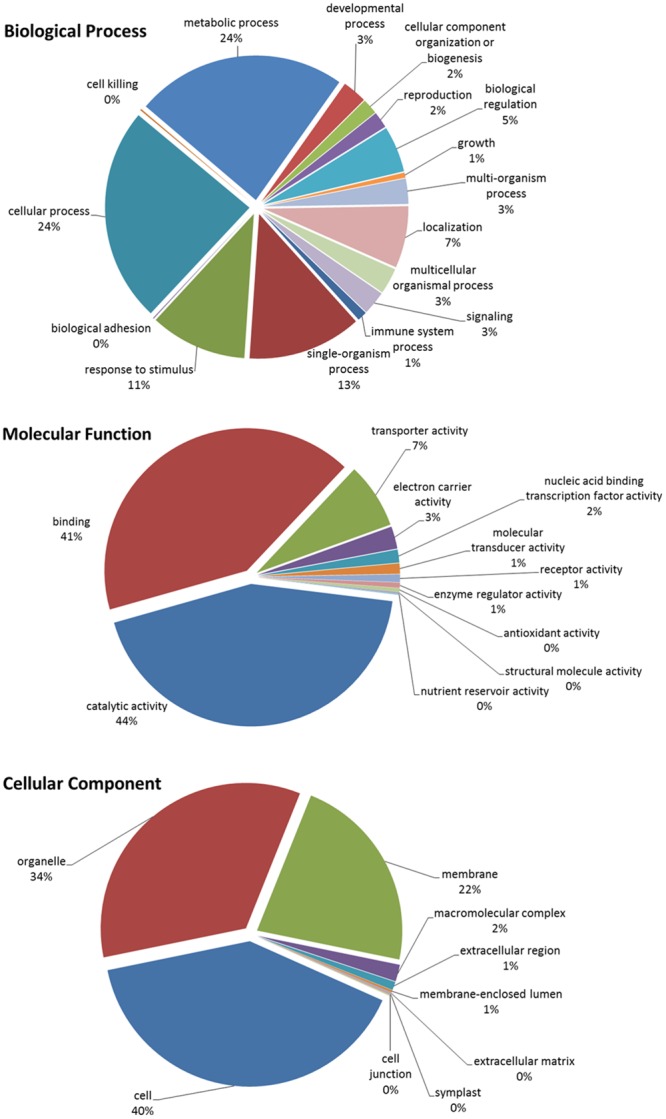
**Blast2GO Level 2 gene ontologies of biological process, molecular function, and cellular component for the total up-regulated transcripts in inoculated and uninoculated resistant buffalograss lines compared with inoculated and uninoculated susceptible lines, respectively.** Of a total of 1356 differentially up-regulated transcripts, 528 were annotated by Blast2GO.

In total, there were 1,356 unique differentially expressed up-regulated transcripts (BUE and URI). These 1,356 transcripts were subjected to Blast2GO and 678 had blast hits resulting in the annotation of 528 transcripts while the other 828 were not annotated by Blast2GO. However, some of the 150 (678 blast hits - 528 annotated sequences) sequences that had blast hits but were not annotated did have assigned protein descriptions that were associated with disease resistance. Among the 528 annotated sequences, 381 sequences had biological process associated gene ontology (GO) terms, 425 sequences had molecular function associated GO terms, and 341 sequences had cellular component associated GO terms. The level 2 GO terms for biological process, molecular function, and cellular component are summarized in **Figure 4**. The most prevalent biological process GOs were cellular process (289 sequences), metabolic process (283 sequences), single-organism process (153 sequences), and response to stimulus (130 sequences). The largest molecular function gene ontologies are catalytic activity (293 sequences) and binding (278 sequences). Cell (301 sequences), organelle (257 sequences), and membrane (166 sequences) are the three largest cellular component gene ontologies.

Additionally, there were a total of 568 unique down-regulated transcripts common to resistant lines (DRI and BDE sets) and they had 209 Blast2GO annotations. Annotated sequences had 152 sequences associated with biological process GO terms, 164 sequences with molecular function GO terms, and 146 sequences with cellular component GO terms. The level 2 GO terms for biological process, molecular function, and cellular component are summarized in Supplementary Figure S1. The most common level 2 GO terms were similar to the ones mentioned in the up-regulated transcripts (BUE and URI) with 49 sequences representing plant defense related GO term response to stimulus.

**FIGURE 5:**
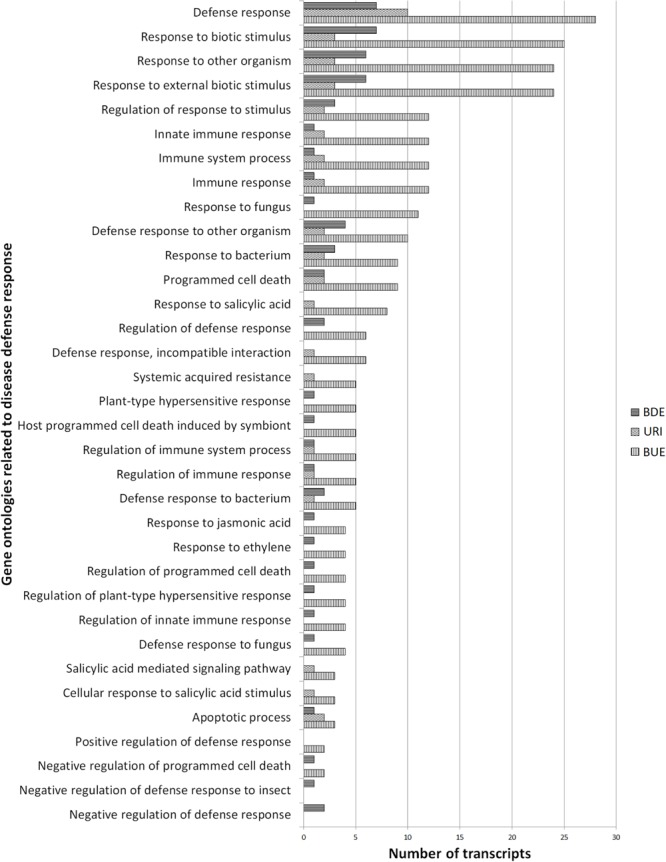
**Number of differentially regulated transcripts associated with host defense response.** The URI group represents the up-regulated transcripts in inoculated resistant buffalograss lines compared to inoculated susceptible lines. The BUE group refers to the transcripts expressed more in uninoculated resistant lines in comparison to uninoculated susceptible lines. BDE represents the down-regulated transcripts in uninoculated resistant lines compared to uninoculated susceptible lines.

Sequences annotated with plant defense related GO terms were searched and selected ontologies are depicted in **Figure 5**. Level 4 and above defense related GO terms with the highest number of associated transcripts included defense response (43 transcripts), response to other organism (33 transcripts), response to external biotic stimulus (33 transcripts), and innate immune response (14 transcripts). These defense related terms are in the biological process GO and were represented by 57 unique transcripts and each transcript was associated with multiple GO terms. These multiple GO terms and the description of proteins encoded by the above 57 sequences are provided in Supplementary Table S2.

Transcripts were identified that shared similarity to known defense response genes. For example, five transcripts (Bodac2015c170619, Bodac2015c160020, Bodac2015c000447, Bodac2015c139533, and Bodac2015c153835) encode ABC transporter-like proteins and have potential to confer non-host resistance (Shimizu et al., 2010). These transcripts were expressed more in leaf spot resistant lines compared (URI, BUE, DRI, and BDE) to the susceptible lines. The transcript Bodac2015c185871 is similar to a bacterial blight resistance gene. Transcripts Bodac2015c098349, Bodac2015c142490, Bodac2015c170497, Bodac2015c106585, and Bodac2015c139347 were up-regulated in resistant lines and similar to genes encoding *Verticillium* wilt disease resistance proteins. Transcript Bodac2015c141715 found in BUE group was homologous to the gene encoding the immediate-early fungal elicitor protein CMPG1. This had been reported to confer a hypersensitive response (HR) in many plants (González-Lamothe et al., 2006).

We identified a LysM domain receptor-like kinase (Bodac2015c159804) along with several (Bodac2015c146460, Bodac2015c164725, Bodac2015c161960, Bodac2015c142835, and Bodac2015c195842) mitogen-activated protein (MAP) kinase and MAP kinase family sequences. Similar genes were reported to cause pattern triggered immunity (PTI) in rice (Shimizu et al., 2010; Balmer et al., 2013). The transcript Bodac2015c130002 was identified among the BUE transcripts and is similar to *Xa21* which mediates resistance against *Xanthomonas* bacteria in rice (Lee et al., 2009).

Nucleotide-binding site leucine rich repeat (NBS-LRR) like transcripts Bodac2015c176239, Bodac2015c100000, and Bodac2015c134520, which encode nb-arc domain proteins, had higher expression in resistant lines. Eighteen leucine-rich repeat receptor-like protein kinase family transcripts were also expressed more in resistant lines. In addition, 16 RPM1-like disease resistance transcripts and one RPS2-like disease resistance transcript (Bodac2015c147648) were identified. The RPM1-like and RPS2-like disease resistance genes have been reported to confer effector-triggered immunity (ETI) in other plants (Mackey et al., 2002; Jones and Dangl, 2006; Balmer et al., 2013). One transcript (Bodac2015c098081) that was similar to the wheat stripe rust resistance gene *Yr10* and the transcripts Bodac2015c145339 and Bodac2015c146056 were similar to the barley stem rust resistant gene *Rpg1* had higher expression in buffalograss leaf spot resistant lines. The *Yr10* and *Rpg1* genes are also responsible for ETI (Brueggeman et al., 2002; Balmer et al., 2013; Zhang et al., 2013).

The transcripts Bodac2015c139945 and Bodac2015c165851 had higher expression (*P* < 0.006) in leaf spot resistant buffalograss lines and encode a heat shock transcription factor-like protein. Heat shock proteins can fold NBS-LRR proteins and make them active against pathogens (Balmer et al., 2013). Nine transcripts with response to salicylic acid GO term were also identified in leaf spot resistant buffalograss lines. All these were found in up-regulated transcript sets URI and BUE. We also found three transcripts (Bodac2015c146262, Bodac2015c159389, and Bodac2015c130171) encoding pathogenesis-related (PR) proteins with higher expression in the resistant lines.

Although this study was not designed to identify pathogen related genes, we searched for genes expressed by *C. inaequalis* since pathogen encoded RNA may have been included in our sequencing reads. The transcript Bodac2015c163958 showed homology to gene encoding NEP1 effector which is a phytotoxic protein identified in *Botrytis cinerea* (Oliver and Solomon, 2010).

### Gene Expression Validation by RT-PCR

**FIGURE 6:**
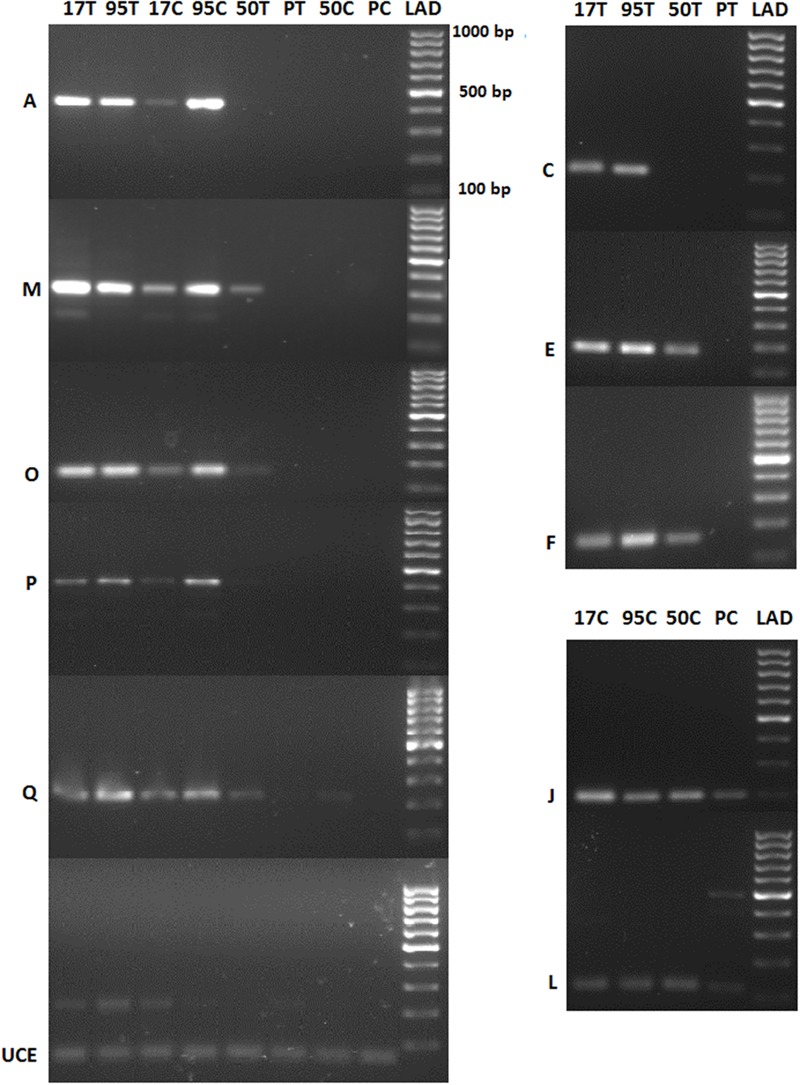
**PCR expression analysis of selected differentially expressed buffalograss genes.** 17, 50, 95, and P refer to NE BG 7-3459-17, NE BG 7-3453-50, 95–55 and Prestige, respectively. C and T followed by the buffalograss lines indicate untreated and treated samples. Ubiquitin conjugating enzyme (*UCE*) is the endogenous gene and positive control. A, M, O, P, and Q are genes which had >10 read counts for both treated and untreated resistant buffalograss lines (95T, 17T, 95C, and 95C) and zero read counts for all susceptible lines (PT, 50T, PC, and 50T). The read counts of treated resistant lines were up-regulated in C, E, and F compared to the treated susceptible lines. J and L genes had higher read counts in untreated resistant lines compared to the untreated susceptible lines. LAD is the molecular marker.

Reverse transcription PCR could distinguish most of the differentially up-regulated genes based on host susceptibility (**Figure 6**). When the difference of gene expression from the transcriptional profiling study was not high, the intensity of PCR bands was similar across all samples (e.g., J and L genes of untreated resistant vs. untreated susceptible samples in **Figure 6** and Supplementary Table S1). The primers used to amplify the endogenous gene *UCE* resulted in a PCR product of the expected size (61 bp) that was uniformly expressed across all samples.

## Discussion

This study was designed to identify buffalograss defense related genes contributing to host resistance against leaf spot disease. We compared inoculated leaf spot resistant and susceptible buffalograss lines to identify genes with either higher or lower expression in the resistant lines. We also identified differentially expressed transcripts in uninoculated resistant lines when compared to uninoculated susceptible lines. Previous research has demonstrated higher basal expression of defense genes in buffalograss lines resistant to chinch bugs (*Blissus occiduus* Barber) compared to susceptible lines (Ramm et al., 2013). Higher basal gene expression may also play a role in buffalograss defense against leaf spot disease.

The ABC transporter like proteins have been reported to confer non-host resistance in *Arabidopsis* against the non-adapted pathogen *Blumeria graminis* f. sp. *hordei* (Stein et al., 2006). Genes conferring non-host resistance don’t produce HR but are normally involved in rapid production of cell wall appositions (physical barriers) and antimicrobial metabolites at the site of pathogen entry (Jones and Dangl, 2006). Infection by *C. inaequalis* has also induced expression of the ABC transporter-like genes Bodac2015c170619, Bodac2015c160020, Bodac2015c000447, Bodac2015c139533, and Bodac2015c153835 in leaf spot resistance lines compared to the susceptible lines.

During the early stages of a pathogen attack the innate immunity of the host allows the plant to overcome the pathogen. In the first stage of this type of immunity, the microbe-associated molecular patterns (MAMP) such as chitin or flagellin are recognized by membrane-localized pattern-recognition receptors (PRR) in plants (Balmer et al., 2013) which results in PTI. In the model monocot rice, LysM PRRs CEBiP and CERK1 have been identified for sensing MAMP chitin (Shimizu et al., 2010). MAMP-signaling activates mitogen-activated protein kinase (MAPK) cascades, which regulate transcription factors (TFs) driving the expression of defense genes (Balmer et al., 2013). In these buffalograss samples we also found genes that encode both LysM domain receptor-like kinase and MAP kinase family proteins. These genes may be involved in PTI in buffalograss against *C. inaequalis*.

The *Xa21* homolog found in buffalograss was originally reported in rice mediating resistance to *Xanthomonas* bacteria. This gene encodes extracellular and intracellular receptors which can sense the 194-amino acid bacterial protein Ax21 and is conserved across all known *Xanthomonas* strains (Lee et al., 2009). During an attack, XA21 induces downstream defense mechanisms which lead to the expression of pathogenesis-related (*PR*) genes and the development of HR (Tena et al., 2011). *Xa21* homologs have been found in other grasses such as *Brachypodium*, sorghum, and maize (Tan et al., 2012). Another example of PTI is the B-lectin receptor kinase Pi-d2 which confers resistance against *Magnaporthe grisea* in rice (Chen et al., 2006). We found 15 transcripts similar to lectin-domain containing receptor kinases in leaf spot resistant buffalograss.

To overcome PTI related defense signaling, pathogens release avirulence (Avr) proteins. In the host, a second line of plant immunity takes place mostly in the cytoplasm and is mediated by NBS-LRR proteins encoded by plant resistance (*R*) genes. These NBS-LRR proteins can recognize and neutralize Avr proteins/effectors which results in ETI (Elmore et al., 2011), and is usually manifest in a HR (Balmer et al., 2013). The NBS-LRR family represents one of the largest and widely conserved gene families in plants. More than one hundred NBS-LRR genes have been identified in the majority of sequenced plants (Balmer et al., 2013). NBS-LRR proteins usually consist of an N-terminal domain with Toll/Interleukin-1 Receptor (TIR-NBS-LRR, or TNL) or an N-terminal coiled-coil (CC-NBS-LRR, or CNL) motif (Meyers et al., 2003). In *Arabidopsis*, RPM1 is a peripheral plasma membrane NBS-LRR protein and in the absence of RPM1, AvrRpm1 effector protein of some *Pseudomonas syringae* strains can cause virulence (Mackey et al., 2002; Jones and Dangl, 2006). RPS2 has been reported to confer ETI to *Arabidopsis* against *P. syringae* (Jones and Dangl, 2006). We identified several NBS-LRR type genes along with genes that encode an RPM1 and RPS2-like disease resistance proteins.

The *Yr10* and *Rpg1* homologs found in leaf spot resistant buffalograss lines confer disease resistance in other graminaceous plants. Presently, 53 different *Yr* genes (*Yr1*–*Yr53*) have been identified to cause stripe rust (*Puccinia striiformis* f. sp. *tritici*) resistance in wheat (Zhang et al., 2013). The barley *Rpg1* gene regulates resistance against the stem rust pathogen *P. graminis* f.sp. *tritici* (Brueggeman et al., 2002). Both of these gene products interact with pathogen effector proteins and confer resistance. The stripe rust resistance protein yr10 and disease resistance protein rpg1 were identified by the Blast2GO analysis. Similarly, many transcripts encoding NBS-LRR type defense related proteins were also identified. However, a search for transcripts that encode proteins similar to TNL and CNL using a hidden Markov model (HMM; Eddy, 1998; Meyers et al., 2003) may reveal more NBS-LRR genes and this analysis is underway.

We found two heat shock like genes (Bodac2015c139945 and Bodac2015c165851) in leaf spot resistant lines. The NBS-LRR proteins activated by heat shock proteins interact with pathogen effectors and regulate WRKY TFs to confer plant resistance (Balmer et al., 2013). WRKY TFs regulate many plant processes including response to biotic stresses (Zhang and Wang, 2005). Plants under pathogen attack can also show enhanced defense activity in tissues not yet attacked through systemic acquired resistance (SAR). When leaf pathogens show localized infection, defense signals are mobilized to distal plant tissues and induce systemic resistance against a broad range of pathogens including fungi, bacteria, viruses, nematodes, and even insects (Shah, 2009). Prior activation of defense genes in distal tissues renders them more resistance against future attacks. In dicots, salicylic acid has been found to play a major role in regulating SAR followed by the up-regulation of pathogenesis-related (*PR*) genes (Balmer et al., 2013). Compared with dicots, the knowledge of SAR in monocots is scarce (Balmer et al., 2013). It is interesting that we identified nine transcripts with “response to salicylic acid” GO term in leaf spot resistant buffalograss lines.

We validated the expression of 10 differentially expressed genes (Supplementary Table S1) by RT-PCR. The majority of the tested genes did produce PCR bands with different intensities and could distinguish resistant and susceptible samples confirming the accuracy of our analysis (**Figure 6**). Quantitative real-time RT-PCR may reveal expression differences of these potential molecular markers more accurately. Interestingly, we found 15 transcripts that have more than 10 read counts in treated and untreated resistant buffalograss and no read counts in the susceptible lines. They may be useful for identifying leaf spot resistant buffalograss lines by molecular methods.

We identified several differentially expressed transcripts that may regulate leaf spot resistance in buffalograss. In particular, the above mentioned 15 sequences that were uniquely expressed in the resistant lines are a new resource for identifying leaf spot resistant buffalograss lines. We also found 21 transcripts in resistant lines that are similar to genes regulating PTI (e.g., sequences encoding LySM, MAPK, lectin receptor kinase-like proteins) and 20 sequences predicted to encode NBS-LRR proteins RMP1, RPS2, RPG1, and YR10 regulating ETI. There were also nine up-regulated transcripts in resistant lines that have potential to initiate SAR and three transcripts encoding PR proteins. This is the first study characterizing changes in the buffalograss transcriptome when challenged with leaf spot pathogen *C. inaequalis*. The NBS-LRR type defense related genes identified here are useful for screening large numbers of buffalograss germplasm for *C. inaequalis* resistance by molecular techniques thus eliminating laborious and time consuming traditional greenhouse and field testing. In addition, buffalograss is more susceptible to *Curvularia* and *Bipolaris* patch diseases when grown in humid regions. These new molecular resources would improve the efficiency for breeding for leaf spot resistant buffalograss cultivars and may help expand the buffalograss into areas prone of leaf spot outbreaks.

## Author Contributions

All authors listed, have made substantial, direct and intellectual contribution to the work, and approved it for publication.

## Conflict of Interest Statement

The authors declare that the research was conducted in the absence of any commercial or financial relationships that could be construed as a potential conflict of interest.

## Footnotes

1 http://www.bioinformatics.babraham.ac.uk/projects/fastqc/

2 http://www.perl.org

3 http://bioinfo.ut.ee/primer3/

4 http://www.ncbi.nlm.nih.gov/bioproject/
