# Supplementary figures and images for: Transcriptome Profiling of Buffalograss Challenged with the Leaf Spot Pathogen Curvularia inaequalis
Source: Front Plant Sci. 2016 May 25;7:715. doi: 10.3389/fpls.2016.00715 (PMC4879344; doi:10.3389/fpls.2016.00715)

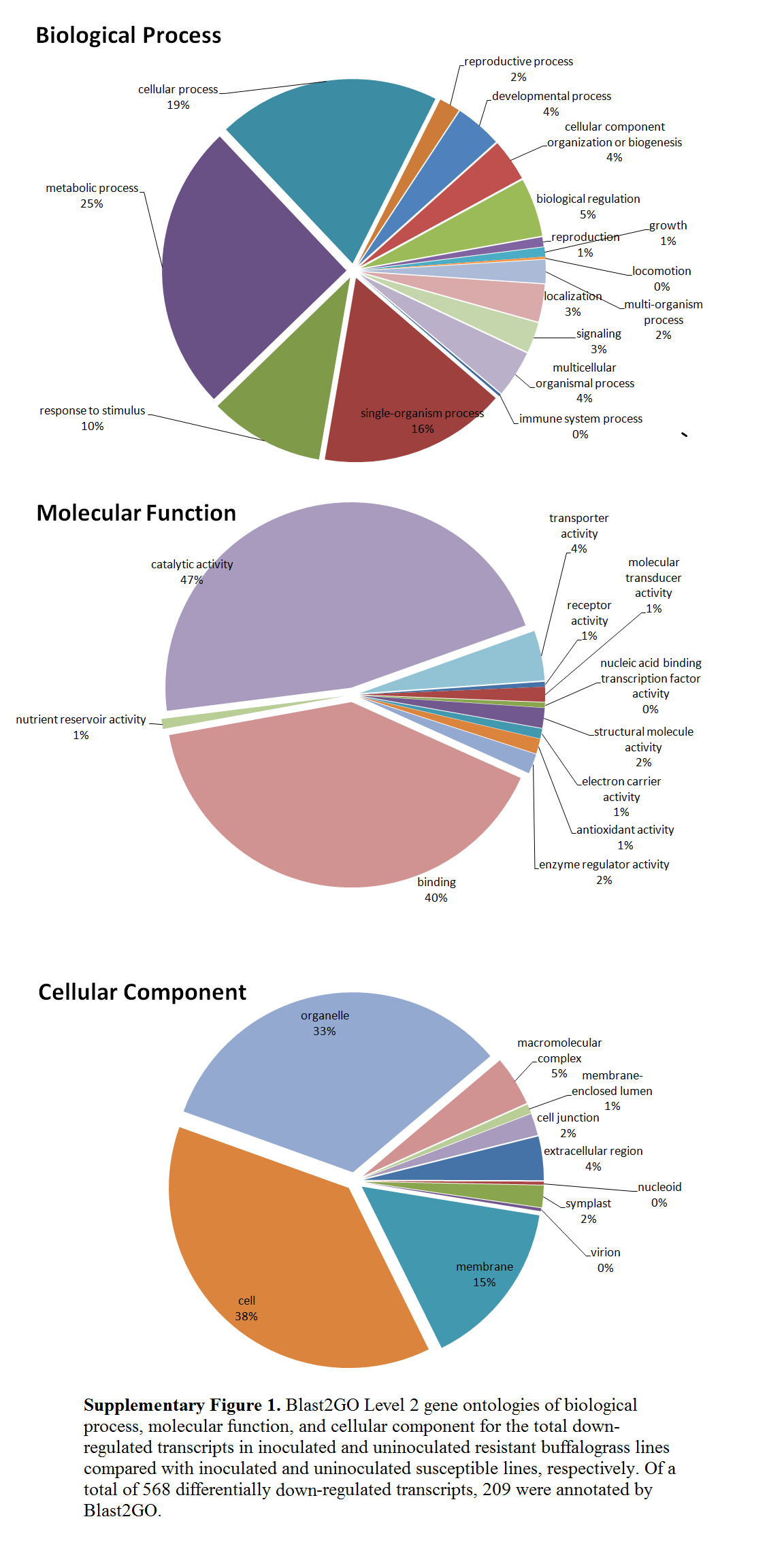

Supplement: Supplementary file 3 [file Image_1.TIF]
